# Depletion of C12orf48 inhibits gastric cancer growth and metastasis via up-regulating Poly r(C)-Binding Protein (PCBP) 1

**DOI:** 10.1186/s12885-022-09220-0

**Published:** 2022-01-31

**Authors:** Lele Lin, Hongbo Li, Dike Shi, Zhiqiang Liu, Yunhai Wei, Wei Wang, Dan Wu, Baozhong Li, Qingqu Guo

**Affiliations:** 1 Department of Gastrointestinal Surgery, the Second Affiliated Hospital of Zhejiang University School of Medicine, 88# Jiefang RoadZhejiang Province, Hangzhou City, 310000 P. R. China; 2 Department of General Surgery, Anyang Tumor Hospital, 1# North Huanbin Road, Henan Province 455000 Anyang City, PR China; 3 Department of Gastrointestinal Surgery, Huzhou Central Hospital, 198# Hongqi RoadZhejiang Province, Huzhou City, 31300 P. R. China; 4 Department of Gastrointestinal Surgery, Shaoxing People’s Hospital, 568# North Zhongxing RoadZhejiang Province, Shaoxing City, 312000 P. R. China

**Keywords:** C12orf48, PCBP1, Gastric cancer, Metastasis, Growth

## Abstract

**Background:**

Gastric cancer remains a major cause of cancer-related death worldwide. C12orf48, also named PARP1 binding protein, is over-expressed in several cancers. However, the expression profile and potential roles of C12orf48 in gastric cancer are largely unknown.

**Methods:**

We used bioinformatics approaches and tissue microarray immunohistochemistry to analyze the expression profile of C12orf48 in gastric cancer tissues. Plasmid-mediated over-expression or knockdown were performed. CCK-8 assays and flow cytometry were employed to evaluate cellular proliferation and apoptosis respectively. Transwell assays were used to assess migrative and invasive abilities. The roles of C12orf48 were also evaluated in a xenograft tumor model.

**Results:**

We found that C12orf48 was over-expressed in gastric cancer tissue, which associated with advanced stage and poor prognosis. In vitro and in vivo experiments showed depletion of C12orf48 attenuated cancer growth, while facilitated apoptosis. Further, the expression of Poly r(C)-Binding Protein (PCBP) 1 was found negatively regulated by C12orf48. Intended up-regulation of PCBP1 prevented C12orf48-mediated proliferation and rescued cells from apoptosis. Besides, C12orf48 promoted cellular migration and invasion, with E-cadherin down-regulated while vimentin and N-cadherin up-regulated, which was reversed by up-regulated PCBP1.

**Conclusions:**

Our findings indicate that depletion of C12orf48 inhibited gastric cancer growth and metastasis via up-regulating PCBP1. Targeting C12orf48-PCBP1 axis may be a potential therapeutic strategy.

**Supplementary Information:**

The online version contains supplementary material available at 10.1186/s12885-022-09220-0.

## Background

Gastric cancer remains the fifth most common malignancy and the third leading cause of cancer-related death worldwide, although the incidence and mortality are gradually on the decrease in most parts of the world in the last decade, except eastern Asia, especially China [1]. Metastatic gastric cancer remains incurable, as the intrinsic genomic mechanism is largely unknown which facilitated cancer cells escaping from current cytotoxic or targeted therapies.

C12orf48, also termed Poly (ADP-Ribose) Polymerase 1 (PARP1) binding protein (PARPBP) or PARI, is a vital negative element of the homologous recombination during DNA repair synthesis [2]. As a PARP1 binding protein, C12orf48 directly binds to PARP1 protein and enhances its activity, which is involved in the DNA damage repair and RNA biogenesis. Hence, it suggests over-expression of C12orf48 might protect cancer cells from cell death induced by DNA damage and has a role in RNA splicing and gene expression [3, 4]. Previous studies have revealed that over-expression of C12orf48 occurred in several cancer types, including myeloid leukemia cells [5], hepatocellular cancer [6], pancreatic cancer [7] and gastric cancer [8]. However, the critical role and molecular basis of altered C12orf48 expression in gastric cancer remain poorly explored.

In the present study, we provided evidence that C12orf48 was much more frequently over-expressed in gastric cancer tissues at both the protein and mRNA level, which was associated with more advanced disease and undesirable median overall survival (mOS). Intended depletion of C12orf48 attenuated cellular proliferation, migration, and invasion, but facilitated apoptosis in vitro. We further found that Poly r(C)-Binding Protein (PCBP) 1, a novel tumor suppressor gene [9], was negatively regulated by C12orf48 downstream. Concurrent over-expression of C12orf48 and PCBP1 abolishes the effects of C12orf48 both in vitro and in vivo. Furthermore, we demonstrated that over-expression of PCBP1 reversed the expression profiles of apoptosis and epithelial mesenchymal transition (EMT) related genes altered by C12orf48. These results highlight C12orf48-PCBP1 signaling contributes to gastric carcinogenesis and provide new insights on gastric cancer growth and metastasis.

## Methods

### Patients and tissues

Patients pathologically diagnosed as gastric cancer and underwent resection were consecutively included at the Department of general surgery, Anyang Tumor Hospital, Anyang City, Henan Province, China, from December 2003 to December 2008. None of these patients received chemotherapy or radiotherapy before resection. Informed consent and study protocol were approved by the Ethics Committee of Anyang Tumor Hospital. Paired gastric cancer tissues and > 5 cm adjacent non-neoplastic gastric epithelium tissues were formalin-fixed, paraffin-embedded and obtained from the Department of Pathology, Anyang Tumor Hospital. Pathological TNM staging was re-evaluated according to the 2009 criteria of the Union for International Cancer Control (UICC). Patients’ general characteristics and survival outcomes were also reviewed retrospectively. All the patients were followed up for overall survival (OS) analysis since resection. Follow-up ingrowth was regularly obtained from outpatient clinical visits and telephone interviews at 3-month intervals. Follow-up was censored when patients’ death or lose of contact. Follow-up was available until December 2009. The mean follow-up time of our patients is 25.1 months (range,1–56 months) [10].

### Cell culture

Human gastric cancer cell line AGS was obtained from the American Type Culture Collection (ATCC, USA). Human gastric cancer cell lines HGC-27 and BGC-823 and Human embryonic kidney epithelial cell line HEK-293 were purchased from the Type Culture Collection Cell Bank (Chinese Academy of Sciences Committee, Shanghai, China). The cells were cultured according to the manufacturer’s instructions. HGC-27 cells were routinely cultured in RPMI-1640 medium (Hyclone, USA) supplemented with 10% Fetal bovine serum (FBS, GIBCO, USA) at 37 °C in a humidified atmosphere of 5% CO_2_. AGS cells were cultured in F-12 K medium (GIBCO, USA). BGC-823 and HEK-293 cells were cultured in DMEM medium (GIBCO, USA).

### Animal experiments

Male nude mice aged 5–8 weeks and weighed 18–20 g (*n* = 21) were purchased from Hangzhou Ziyuan Experimental Animal Technology Co., Ltd by the Animal Ethics Committee of Zhejiang University. We selected a small sample size because the effect of C12orf48 and PCBP1 in vivo was evaluated for the first time in the present study, and therefore, the initial intention was to gather evidence verifying the results of in vitro experiments. The mice were randomly divided into 3 groups, 7 in each group. The tumor-bearing nude mice models were established with (1) HGC-27 cells, (2) C12orf48 over-expression stable transgenic HGC27 cells lines and (3) concurrent C12orf48-PCBP1 over-expression stable transgenic HGC27 cells. The cells were re-suspended in Dulbecco's phosphate buffered saline (DPBS) and counted. 1 × 10^6^ cells were subcutaneously inoculated into the lateral back of the forelimb axilla on day one. The mice weights and transplanted tumors were measured twice a week. Tumor volumes were estimated according to the following formula: V = 1/2*a*b^2^, where a is the long axis and b is the short axis. The mice were sacrificed on day 15. The tumors were dissociated, photographed, and prepared for following Immunohistochemistry, Tunel assays or Western blotting.

### Tissue microarray (TMA) construction

Tissue microarray was constructed as described previously [10]. Briefly, representative areas in the center of each specimen were selected by a pathologist and transferred to a TMA block using a 1.5 mm core diameter needle. H&E-stained sections from each TMA block were checked by a pathologist to ensure that adequate targeted areas had been included. The blocks contained a total of 109 paired tumor tissue samples and non-neoplastic gastric epithelium samples. Four-micrometer-thick sections were cut from each tissue array block for immunohistochemistry study.

### Immunohistochemistry (IHC)

Briefly, the formalin-fixed, paraffin-embedded tissue slides were dewaxed and dehydrated through graded alcohol. The endogenous peroxidase was exhausted with 3% H_2_O_2_ and the slides were boiled and cooled for the antigen retrieval. Next, nonspecific binding was blocked. Then the sections were incubated with primary antibodies (Rabbit polyclonal anti-C12orf48, Rabbit polyclonal anti-Ki-67; Abcam, Cambridge, MA, USA) overnight at 4◦C, and treated with secondary antibodies (Shanghai Changdao Biotechnology Co., Ltd, Shanghai, China). Next, tissue sections were counterstained with hematoxylin, dehydrated, and mounted. Normal goat serum was used as a negative control. The stained sections were reviewed microscopically and evaluated independently by two observers using Image J (v1.8.0). The intensity (negative, weak, moderate, or strong) and the percentage (0%, 1–10%, 11–50%, and > 50%) of positive tumor cells were scored (0, 1, 2, 3). The immunoreactivity score (IRS) was calculated as the sum of staining intensity and percentage. Subsequently, a binary classification was applied, combining IRS scores of 0–3 into ‘negative’ and 4–6 into ‘positive’ expression [10].

### Vectors and transfection

The short hairpin RNA (shRNA) vectors against C12orf48 were generated using oligonucleotides (h-C12orf48-sh1: ACACAGTATCTCCTAGTCA, h-C12orf48-sh2: TGAAGAACAGTAATATGTT, h-C12orf48-sh3: TGATTGATGTTTATCAAAA), which were annealed and introduced into the pHAV3.1-shRNA-tGFP vector (Shanghai Asia-Vector Biotechnology, Shanghai, China). The reconstructed C12orf48 shRNA vectors were then incubated with DH5α cells in LB medium containing ampicillin for 12–16 h, followed by extraction and sequencing from positive clones. A scrambled shRNA was used as control. The Over-expression vectors were generated using Plv304 lentivirus vectors (Shanghai Asia-Vector Biotechnology, Shanghai, China). Gene DNA was amplified by PCR using specific PCR primers. Then agarose gel electrophoresis was blotted, and the gene DNA was retrieved. Next, Plv304 lentivirus vector underwent restriction digestion and ligated to retrieved DNA using Ligation-Independent cloning (LIC) method. The reconstructed expression vectors were then incubated with DH5α cells in LB medium containing ampicillin for 12–16 h. Finally, positive clones were extracted and sequenced. The C12orf48 PCR primers forward: 5’-TTTCCGGTGAATTCATGGCTGTGTTTAATCAGAA-3’, and reverse: 5’-AGAGGGGCGGGATCCTTATAGTCTAAAAAACTGAG-3’. The PCBP1 PCR primers forward: 5’-AAGTTTGTACAAAAAAGTTGGCATGGATGCCGGTGTG-3’, and reverse: 5’-CCGGTTAGCGCTAGCTCATTACTACGTAGAATCGAGAC-3’. Transfection of over-expression or shRNA vectors at a final concentration of 8 ng/μl were using Lipofectamine™ 2000 (Invitrogen, Beijing, China). Cells were incubated in a serum-free medium for 4–6 h, followed in a serum-containing medium for 48 h. The expression profiles of GFP were detected using fluorescence microscope and transfection efficiency was determined using qRT-PCR. Then the cells were collected and subjected to assays.

### Quantitative real time PCR (qRT-PCR)

Total RNA was isolated using Trizol reagent (YEASEN, Shanghai, China) according to the manufacturer’s instruction and stored in DEPC-treated water at -80 ℃. For usage, RNA was bathed and denatured at 70 ~ 85 ℃ for 5 min and ice cold immediately. cDNA then reverse transcribed using TransScript All-in-One First-Strand cDNA Synthesis SuperMix for qPCR kit (TransGen Biotech, Beijing, China). QPCR was performed using NovoStart®SYBR qPCR SuperMix kit (Novoprotein, Shanghai, China). The primers are: h-GAPDH Forward: GATGAGATTGGCATGGCTTT, Reverse: GTCACCTTCACCGTTCCAGT; h-C12orf48 Forward: TGAAACATGCTGCTCGAGAG, Reverse: AGGATCTGATGGTGGTGGTG; h-PCBP1 Forward: GCCGGTGTGACTGAAAGTG; h-PCBP1 Reverse:CCCAATGATGCTTCCTACTTCC.

### CCK-8 assays

The cellular viability and proliferation were detected by CCK-8 assays according to the manufacturer’s instruction (Sangon Biotech, Shanghai, China). Cells were seeded in 6-well plate and grown to 60 ~ 70% confluence. Then transfection of over-expression vectors and shRNA vectors was performed. 12 h after transfection, cells were re-suspended and counted. And 1.5 × 10^4^ cells/well were seeded into 96-well plate. By 60 h or specific times after transfection, CCK-8 reagents were added and incubated for 2 h. Optical density (O.D.) values were then determined by Spectrophotometer (Pattern number: 752, Shanghai Sunny Hengping Scientific Instrument Co., Ltd., Shanghai, China) at 450 nm wave.

### Flow cytometry

Detection of cellular apoptosis was performed with Annexin V-PE/7-AAD apoptosis detection kit according to the manufacturer’s instruction (YEASEN, Shanghai, China). Briefly, 1 × 10^6^ cells were seeded in 6-well plate and grown to 60 ~ 70% confluence. Then over-expression or shRNA vectors were transfected. 72 h after transfection, the cells were incubated with Annexin V-PE and 7-AAD in the dark. Subsequently, cellular apoptosis was analyzed by flow cytometer (Cell Lab Quanta SC, Beckman, USA).

### Transwell assays

Cell migration and invasion assays were performed using transwell assays. Cells suspended in 500 μl medium were seeded in 6-well plate. Transfection of over-expression and shRNA vectors was performed when cells were grown to 60 ~ 70% confluence. 12 h after transfection, cells were re-suspended and counted. And 1.5 × 10^4^ cells/well were seeded into Falcon Cell Culture Inserts (8-μm pore; Corning, CA, USA) for migration assays or Corning BioCoat Matrigel Invasion Chambers (8-μm pore; Corning, CA, USA) for invasion assays. 6 h later, medium was replaced: medium supplemented with 2% FBS was added into the upper chambers, and medium supplemented with 20% FBS was added into the lower chambers of the 24-well plate (Corning, CA, USA) as the chemoattractant. By 60 h after transfection, the non-migrating or non-invading cells were removed from the upper surface of the membrane of the inserts or chambers by cotton swabs. The cells on the lower surface were fixed in 4% paraformaldehyde, stained with Gram’s reagent and air dried. Finally, the cells were counted at a magnification of × 100 in at least three random fields, using an inverted fluorescence microscope (Olympus, Tokyo, Japan) and photographed with a digital camera.

### Western blotting

Total protein was collected 96 h after treatment or transfection. The concentrations of cell lysates were determined using BCA protein assay kit (Beyotime Biotechnology, Shanghai, China). Cell lysates were resolved by SDS-PAGE. The gels were cut according to the precise groups and the proteins were transferred to polyvinylidene fluoride (PVDF) membranes and immunoblotted. Next, the density of each band was measured using Image Quant LAS 500 (GE, USA) and normalized to that of the respective control band. The antibodies included primary antibodies against C12orf48, PCBP1, BAX, BCL-2, Caspase 3, Cleaved Caspase 3, PARP1, Cleaved PARP1, Vimentin, E-cadherin, N-cadherin, GAPDH and α-Tubulin (Abcam, Cambridge, MA, USA), and secondary horseradish peroxidase-conjugated antibodies (Zhong Shan Golden Bridge Biotechnology Co., Ltd, Beijing, China).

### TdT-mediated dUTP Nick-End Labeling (Tunel)

Tunel assays were performed with Tunel kit (Roche, USA) according to the manufacturer’s introduction. Briefly, the tissue slides were dewaxed, rehydrated through graded alcohol, digested with trypsin and washed. Then, the slides were incubated with TUNEL reaction mixture, which contained FITC marked dUTP, in dark. Next, the slides were stained and sealed by DAPI (Beyotime Biotechnology, Shanghai, China) supplemented with anti-quenching mounting medium (Beyotime Biotechnology, Shanghai, China) for fluorescence microscope filming. And rates of apoptosis cells were evaluated by using Image J (v1.8.0).

### Statistical analysis

Statistical analysis was performed with SPSS software (Version 21). The categorical variables were compared by using the Chi-square test. OS was plotted and compared using the Kaplan–Meier method, with differences across groups assessed by the log-rank statistic. The continuous variables between two groups were compared by using the Student's t test. And comparisons among multiple groups were performed by one-way analysis of variance (ANOVA) followed by Bonferroni post hoc test. Experiments were performed in triplicate and repeated at least three times. Data were expressed as mean values ± standard deviation (SD). A *P* value < 0.05 was considered to indicate statistical significance.

## Results

### Over-expression of C12orf48 in gastric cancer tissues related to poor prognosis

**Fig. 1:**
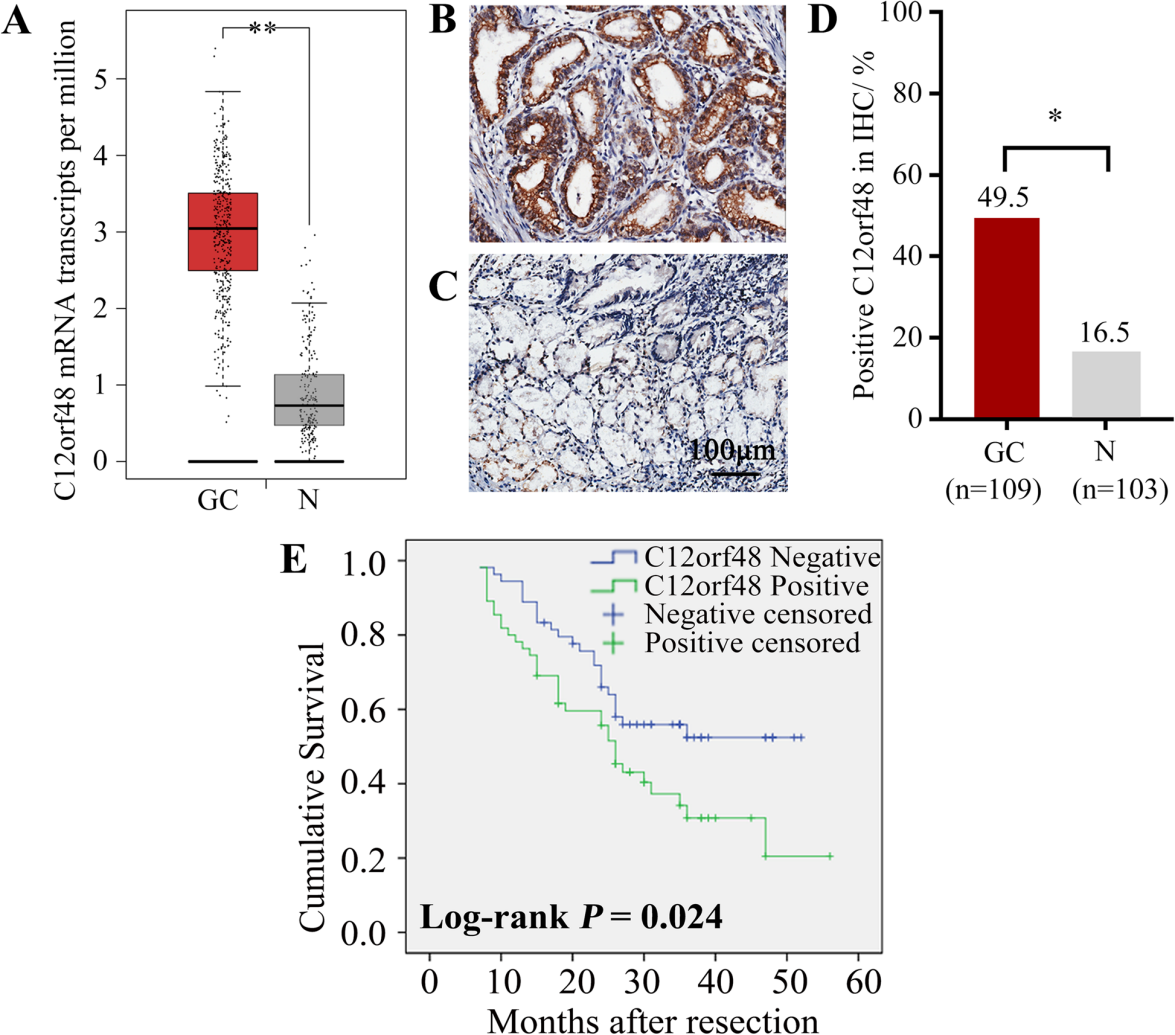
C12orf48 was over-expressed in gastric cancer tissues and associated with undesirable OS. **A** GEPIA data showed the C12orf48 mRNA transcripts per million was significantly up-regulated in gastric cancer tissues (*n* = 408) compared with non-neoplastic gastric epithelium tissues (*n* = 211). **B-C** Paired clinical gastric cancer tissues and non-neoplastic gastric epithelium tissues were immunohistochemically stained (*n* = 109). Representative images showed positive expression of C12orf48 protein in gastric cancer tissue (**B**), while negative expression in the normal one (**C**) (magnification, × 200). **D** Summary of C12orf48 expression in different gastric specimens. **E** Kaplan–Meier estimates of OS. Asterisks (*) indicate a significant difference compared with control groups (* *P* < 0.05, ** *P* < 0.01). GC, Gastric cancer; N, Non-neoplastic gastric epithelium

In order to explore the expression profile of C12orf48 in gastric cancer, we firstly employed Gene Expression Profiling Interactive Analysis (GEPIA, http://gepia.cancer-pku.cn/index.html). GEPIA partly combined The Cancer Genome Atlas (TCGA) and Genotype-Tissue Expression (GTEx) database and analyzed gene expression data from both of gastric cancer tissues (*n* = 408) and non-neoplastic gastric epithelium tissues (*n* = 211). The results demonstrated a significant up-regulation of C12orf48 mRNA transcripts per million in gastric cancer tissues compared with non-neoplastic gastric epithelium tissues (*P* < 0.01) (Fig. 1A). Next, we obtained a total number of 109 paired gastric cancer tissues and non-neoplastic gastric epithelium tissues from patients initially received resection. Immunohistochemistry staining of C12orf48 in both groups was performed to review the expression of C12orf48 at protein level, except 6 of non-neoplastic gastric epithelium specimens because of tissue damage. Figure 1B representing positive results, showed high expression of C12orf48 in gastric cancer tissue, noting the protein abundant in the nuclear, while Fig. 1C showed negative results in non-neoplastic gastric epithelium. Consistent with the GEPIA data, data from retrieved clinical specimens showed that C12orf48 had a significantly higher percentage of expression in gastric cancer tissues (49.5%, 54/109) than that in the non-neoplastic gastric epithelium (16.5%, 17/103) (*P* < 0.05) (Fig. 1D). Taken together, the results suggested that C12orf48 was over-expressed in gastric cancer tissues at both the protein and mRNA level.

Next, to found out whether the altered expression of C12orf48 associated with the prognosis of gastric cancer patients, we collected these patients’ clinicopathological features and survival outcomes. Table 1 showed a significantly irrelevant relationship between C12orf48 over-expression and age, gender, histological subtypes, vessel cancer embolus, pTNM stage, tumor size (all *P* > 0.05), while C12orf48 overexpression was significantly in relate to T3-4 depth of invasion (53.7% v.s. 21.4%, *P* < 0.05), positive perineural invasion (73.7% v.s. 44.4%, *P* < 0.05) and positive nodal metastasis (56.4% v.s. 32.9%, *P* < 0.05) between the two groups. Besides, Kaplan–Meier survival analysis was performed to assess the prognostic value of C12orf48 in gastric cancer. Clearly, poorer likelihood of mOS was observed in gastric cancer patients with high C12orf48 expression (Log-rank *P* < 0.05) (Fig. 1E). Thus, over-expression of C12orf48 might associated with advanced gastric cancer and undesirable mOS.

**Table 1:** Associations between the expression of C12orf48 with clinicopathological features in 109 patients with gastric cancer

**Parameter**	Total (*n* = 109)	Number of cases		*P*-value
		Negative	Positive	
**Age at diagnosis (years)**				
< 60	35	20 (57.1%)	15 (42.9%)	0.413
≥ 60	74	35 (47.3%)	39 (52.7%)	
**Gender**				
Male	87	44 (50.6%)	43 (49.4%)	0.575
Female	22	11 (50.0%)	11 (50.0%)	
**Histological subtypes**				
Well + Moderately differentiated	65	35 (53.8%)	30 (46.2%)	0.438
Poorly differentiated + Signet ring cell	44	20 (45.5%)	24 (54.5%)	
**Depth of invasion**				
T 1 + 2	14	11 (78.6%)	3 (21.4%)	0.042
T 3 + 4	95	44 (46.3%)	51 (53.7%)	
**Vessel Cancer Embolus**				
No	92	48 (52.2%)	44 (47.8%)	0.698
Yes	17	7 (41.2%)	10 (58.8%)	
**Perineural invasion**				
No	90	50 (55.6%)	40 (44.4%)	0.024
Yes	19	5 (26.3%)	14 (73.7%)	
**Nodal metastasis**				
No	31	21 (67.1%)	10 (32.9%)	0.033
Yes	78	34 (43.6%)	44 (56.4%)	
**pTNM stage**				
I/II	39	23 (59.0%)	16 (41.0%)	0.184
III/IV	70	32 (45.7%)	38 (54.3%)	
**Tumour size(cm)**				
< 6	63	32 (50.8%)	31 (49.2%)	1.000
≥ 6	46	23 (50.0%)	23 (50.0%)	

### Depletion of C12orf48 inhibited cellular proliferation, migration and invasion abilities, while facilitated apoptosis of gastric cancer cells

**Fig. 2:**
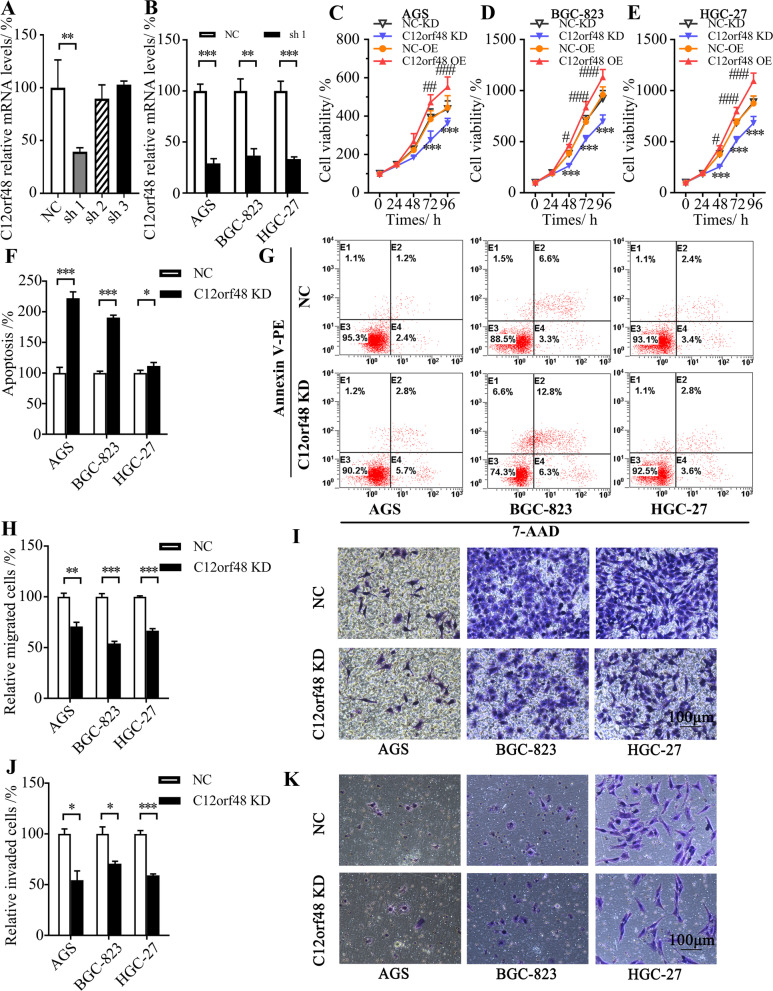
Depletion of C12orf48 inhibited cellular proliferation, migration and invasion abilities, while facilitated apoptosis of gastric cancer cells. **A** Control and C12orf48 shRNA vectors were transfected into HEK-293 cells. The expression of C12orf48 mRNA was determined by qPCR. **B** AGS, BGC823 and HGC27 gastric cancer cell lines were transfected with control or C12orf48 shRNA 1 vector. The expression of C12orf48 mRNA was determined by qPCR. **C-E** AGS, BGC823 and HGC27 gastric cancer cell lines were transfected with C12orf48 shRNA 1 vector, over-expression vector or their respective controls. 60 h after transfection, proliferated cells were subjected to CCK-8 assays to measure O.D. (optical density) values. **F-G** 72 h after transfection, cellular apoptosis was detected with Annexin V-PE and 7-AAD using flow cytometry. And the rates of apoptosis cells were analyzed. **H–K** 12 h after transfection, cells were seeded into transwell inserts or chambers. By 60 h after transfection, the cells were imaged and counted (magnification, × 100). Representative images were captured from the migrated (**I**) and invaded (**K**) cells. The number of migrated (**H**) and invaded (**J**) cells were counted. Experiments were repeated at least three times. Asterisks (*) indicate a significant difference between knockdown group and control group (* *P* < 0.05, ** *P* < 0.01 and *** *P* < 0.001). The hash symbols (#) indicate a significant difference between overexpression group and control group (# *P* < 0.05, ## *P* < 0.01 and ### *P* < 0.001). NC, negative control. KD, knowdown. OE, overexpreesion

Then, we investigated whether altered C12orf48 expression might affect cellular functions of gastric cancer cells. C12orf48 shRNA vectors were constructed to deplete C12orf48 expression. We firstly assessed the three constructed shRNAs in HEK-293 cells. qPCR results revealed that shRNA 1 inhibited the expression of C12orf48 mRNA the most (Fig. 2A). Then, we confirmed that shRNA 1 effectively down-regulated C12orf48 in AGS, BGC823 and HGC27 gastric cancer cell lines (Fig. 2B). Hence, we used shRNA 1 in the following experiments, including cellular proliferation, apoptosis, migration, and invasion investigations. The results showed that depletion of C12orf48 significantly harmed cell proliferation in gastric cancer cell lines, including AGS, BGC-823 and HGC-27 (Fig. 2C-E) and contributed to apoptosis (Fig. 2F-G) (*P* < 0.05). On the other side, intended up-regulation of C12orf48 promoted cell proliferation in these cell lines further (Fig. 2C-E). Besides, cell migration (Fig. 2H-I) and invasion (Fig. 2J-K) abilities were assessed by transwell assays. Compared with control groups, the numbers of migrated and invaded cells were markedly decreased in C12orf48 depleted groups (*P* < 0.05).

### Depletion of C12orf48 up-regulated the expression of PCBP1, which associated with improved mOS

**Fig. 3:**
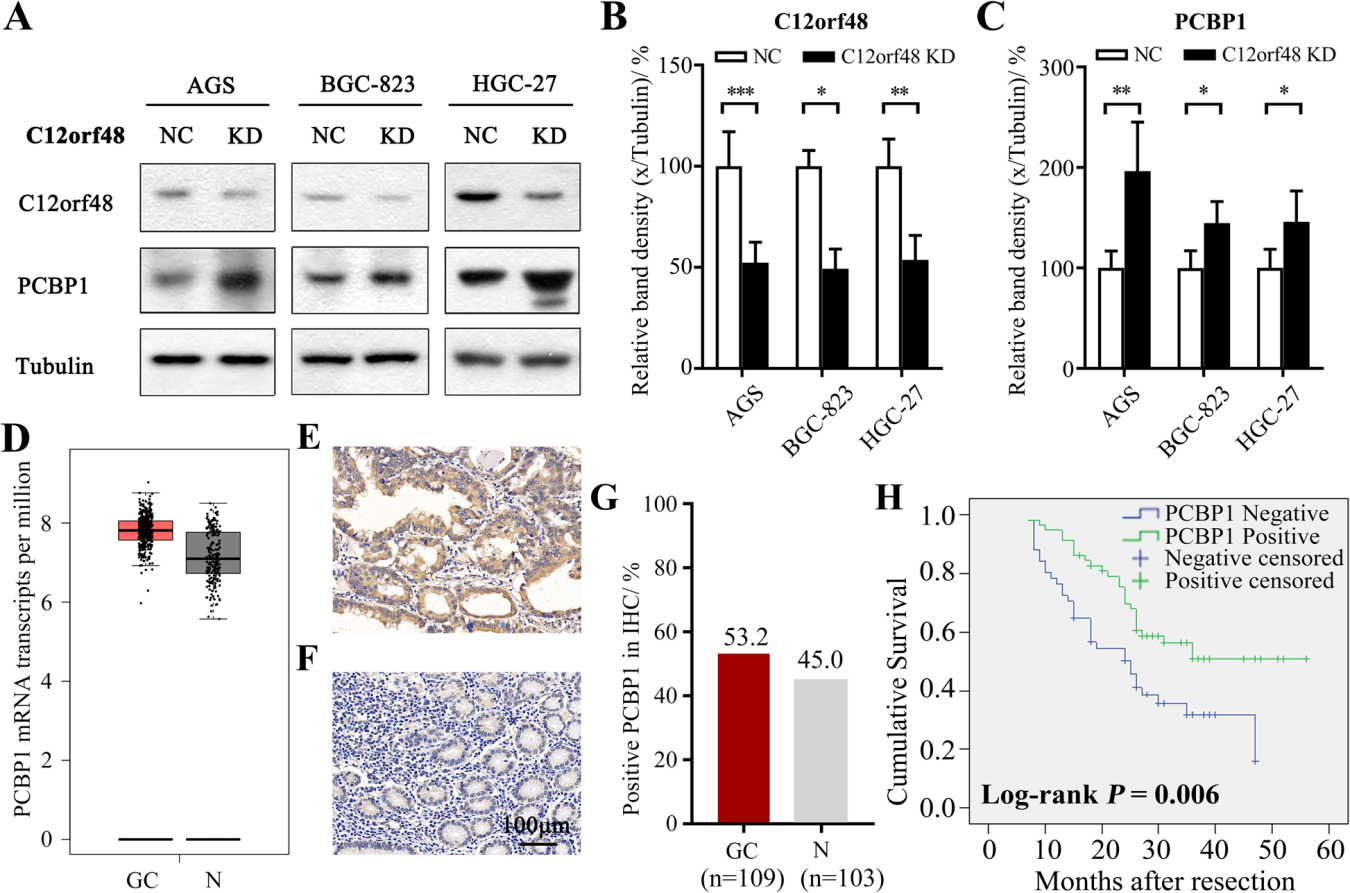
C12orf48 negatively relates to PCBP1 expression. AGS, BGC823 and HGC27 gastric cancer cell lines were transfected with control or C12orf48 shRNA 1 vector. **A** Cells were subjected to western blotting 96 h after transfection. **B-C** The density of each C12orf48 band (**B**) and each PCBP1 band (**C**) was measured and normalized to that of the respective Tubulin band. **D** GEPIA data showed the PCBP1 mRNA transcripts per million was comparable between gastric cancer tissues (*n* = 408) and non-neoplastic gastric epithelium tissues (*n* = 211). (**E–F**) Paired clinical gastric cancer tissues and non-neoplastic gastric epithelium tissues were immunohistochemically stained (*n* = 109). Representative images showed positive expression of PCBP1 protein in gastric cancer tissue (**E**), while negative expression in the normal one (**F**) (magnification, × 200). **G** Summary of PCBP1 expression in different gastric specimens. (**H**) Kaplan–Meier estimates estimated of the correlation between OS and PCBP1 expression. Experiments were repeated at least three times. Asterisks (*) indicate a significant difference compared with control groups (* *P* < 0.05, ** *P* < 0.01 and *** *P* < 0.001). NC, negative control. KD, knowdown

**Fig. 4:**
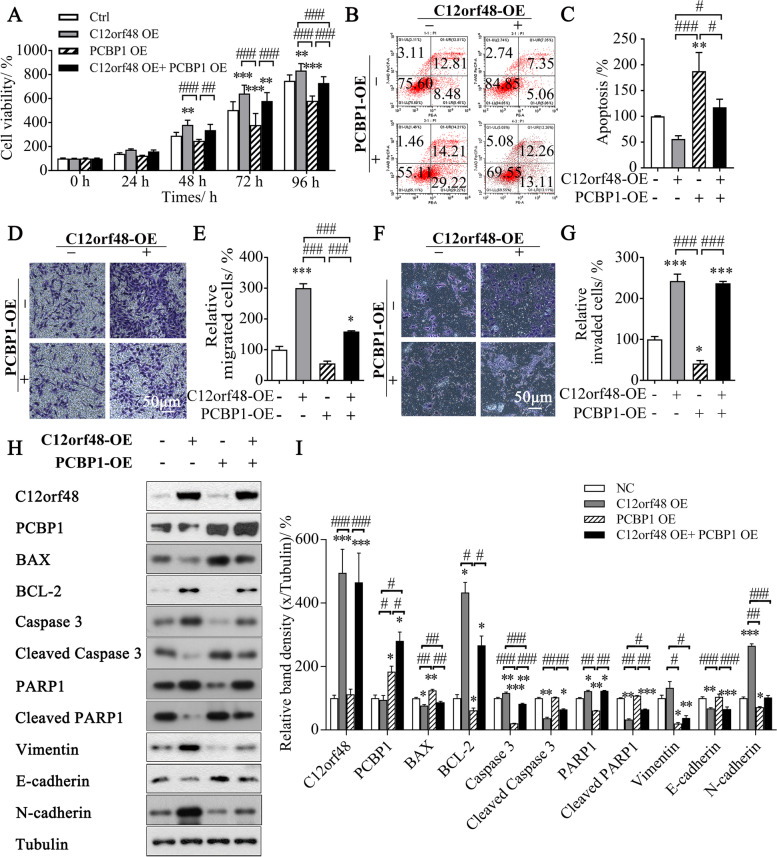
Over-expression of PCBP1 abolishes the effects of C12orf48 in gastric cancer cells. HGC27 gastric cancer cells were transfected with control, C12orf48 over-expression vector or concurrent C12orf48 and PCBP1 over-expression vectors. **A** Proliferated cells were subjected to CCK-8 assays to measure O.D. values at 0, 24, 48, 72, 96 h after transfection. **B** 72 h after transfection, cellular apoptosis was detected with Annexin V-PE and 7-AAD using flow cytometry. **C** The rates of apoptosis cells were analyzed. **D-G** 12 h after transfection, cells were seeded into transwell inserts or chambers. By 60 h after transfection, representative images were captured from the migrated (**D**) and invaded (**F**) cells (magnification, × 100). And the numbers of migrated (**E**) and invaded (**G**) cells were counted and compared. **H** 96 h after transfection, the cells were subjected to western blotting. **I** The density of each band was measured and normalized to that of the respective Tubulin band. Experiments were repeated at least three times. Asterisks (*) indicate a significant difference compared with control groups (* *P* < 0.05, ** *P* < 0.01 and *** *P* < 0.001). The hash symbols (#) indicate a significant difference between specified groups (# *P* < 0.05, ## *P* < 0.01 and ### *P* < 0.001). NC, negative control. KD, knowdown. OE, overexpression

Previous studies reviewed that C12orf48 has a role in DNA damage and RNA expression. Next, we performed western blotting in AGS, BGC823 and HGC27 cell lines to screen the potential molecules downstream C12orf48. Fortunately, we found that intended knockdown of C12orf48 by shRNA markedly up-regulated the expression of PCBP1, which was involved in the process of pre-mRNA, mRNA stabilization and translation and was validated responsible for cancer growth and metastasis functioning as a novel tumor-suppressive factor^9^ (Fig. 3A-C). While over-expression of C12orf48 by using overexpressed vectors had little effect on the expression level of PCBP1 (Fig. 4H).

In view of this, we investigated whether the expression profile of PCBP1 was altered in gastric cancer tissues and its correlation with the clinical significance. We also employed the GEPIA website and TMA immunohistochemistry to analysis the expression profile of PCBP1 between gastric cancer tissues and non-neoplastic gastric epithelium tissues. The results showed the expression levels of PCBP1 were comparable between the two groups (*P* > 0.05) (Fig. 3D-G). Even so, the Kaplan–Meier survival analysis demonstrated that improved mOS was observed in gastric cancer patients with high PCBP1 expression (Log-rank *P* < 0.05) (Fig. 3H).

### Over-expression of PCBP1 abolishes the effects of C12orf48 in gastric cancer cells

Further, we over-expressed PCBP1 to identify whether PCBP1 had a role in C12orf48-mediated cellular processes. Over-expression of C12orf48 facilitated cellular proliferation 48 h after transfection, while over-expression of PBCP1 attenuated cellular proliferation and promoted apoptosis. Concurrent over-expression of C12orf48 and PBCP1 significantly harmed the proliferation-promoting effect mediated by C12orf48 96 h after transfection, and slightly protected cells from apoptosis (Fig. 4A-C). As for cell metastasis ability, intended over-expression of PCBP1 tended to inhibit cell migration and invasion. Concurrent over-expression of C12orf48 and PBCP1 could significantly reverse C12orf48-mediated migration, but little effect on C12orf48-mediated invasion (Fig. 4D-G).

### Downstream signaling of C12orf48-PCBP1 axis

We also examined the downstream signaling of C12orf48-PCBP1 axis. The anti-apoptosis Bcl-2 was up-regulated by C12orf48 over-expression and partially reversed by PCBP1. On the other side, the pro-apoptosis BAX and cleaved caspase 3 were down-regulated by C12orf48 over-expression and rescued by PCBP1. Besides, we found that EMT-related protein Vimentin and N-cadherin, the mesenchymal markers, was up-regulated by C12orf48, and markedly down-regulated by concurrent over-expression of C12orf48 and PCBP1. However, E-cadherin, an epithelial marker, was down-regulated by C12orf48 and reversed by PCBP1 (Fig. 4H-I).

### Over-expression of PCBP1 reverses the effects of C12orf48 in vivo

**Fig. 5:**
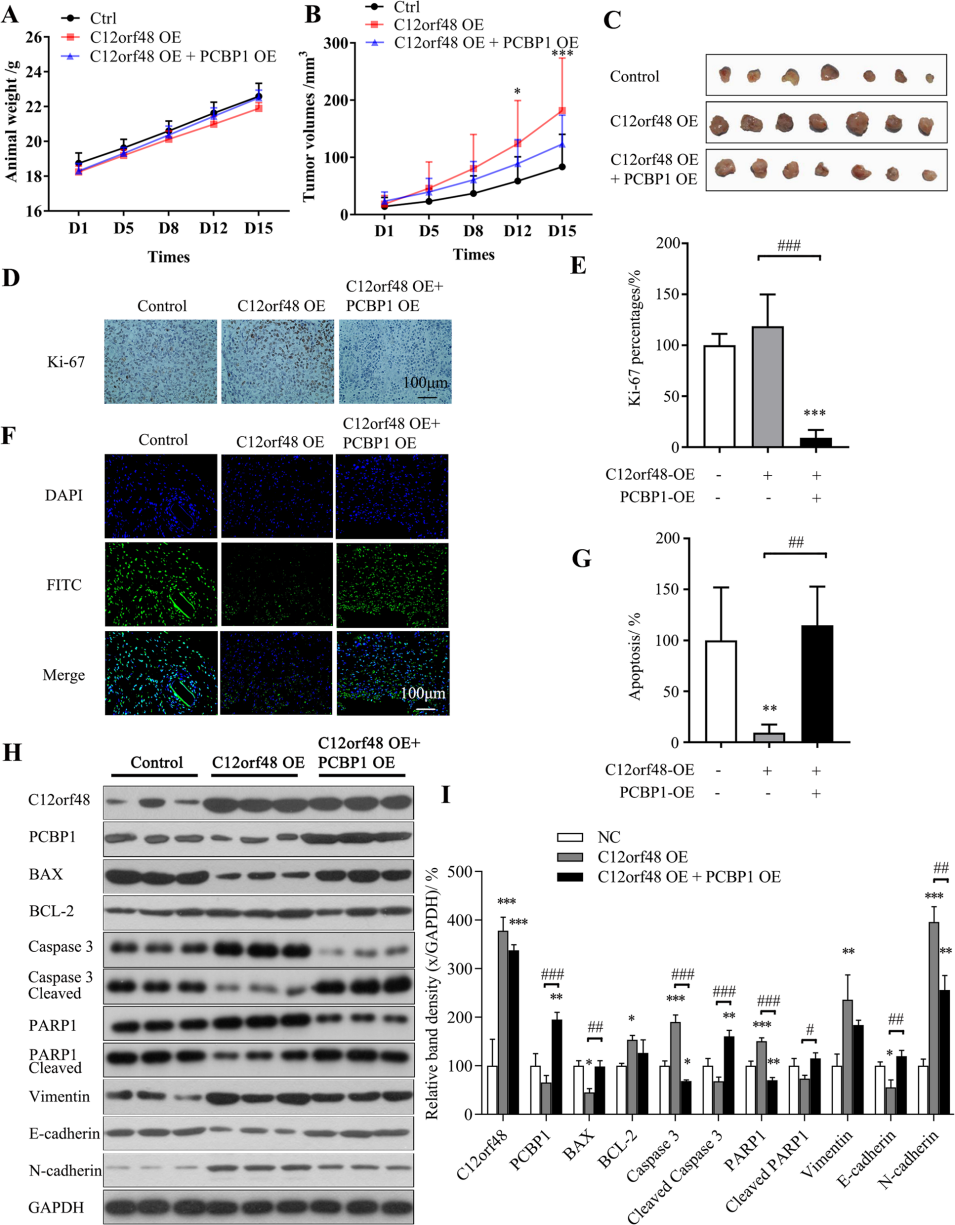
Over-expression of PCBP1 attenuates the effects of C12orf48 in vivo. The tumor-bearing nude mice were established with HGC27 cells, C12orf48 over-expression stable transgenic HGC27 cells lines and concurrent C12orf48-PCBP1 over-expression stable transgenic HGC27 cells, respectively. Animal weights (**A**) and tumor volumes (**B**) were recorded from day 1 to day 15. On day 15, the mice were sacrificed, and the tumors were dissociated and prepared for subsequent assays. **C** The dissociated tumors were photographed. **D** Representative Immunohistochemistry images of Ki-67, a proliferative marker, were captured (magnification, × 200). **E** Ki-67 percentages were compared. **F** Representative images of Tunel assays were captured (magnification, × 200). Cellular nuclei were stained with DAPI (blue) and the apoptotic cells were stained with FITC (green). **G** The rates of cell apoptosis were compared. **H** Protein of tumors were extracted and subjected to western blotting. **I** The density of each band was measured and normalized to that of the respective GAPDH band. Experiments were repeated at least three times. Asterisks (*) indicate a significant difference compared with control groups (* *P* < 0.05, ** *P* < 0.01 and *** *P* < 0.001). The hash symbols (#) indicate a significant difference between specified groups (# *P* < 0.05, ## *P* < 0.01 and ### *P* < 0.001). NC, negative control. OE, overexpression

For *in-vivo* experiments, we established tumor-bearing nude mice models with HGC27 cells, C12orf48 over-expression stable transgenic HGC27 cells lines and concurrent C12orf48-PCBP1 over-expression stable transgenic HGC27 cells. Animal weights and tumor volumes were recorded with time (Fig. 5A-B). Xenograft tumor volumes were significantly increased in C12orf48 OE group compared with the control group, while concurrent C12orf48-PCBP1 over-expression partly shrunk the transplanted tumors (Fig. 5B). The photos of dissociated tumors were presented (Fig. 5C). Also, the percentage of positive Ki-67 expression was markedly up-regulated in C12orf48 OE group and reversed by concurrent C12orf48-PCBP1 over-expression (Fig. 5D-E). On the other side, Tunel assays showed over-expression of C12orf48 attenuated cellular apoptosis process compared with the control group while PCBP1 over-expression enhanced apoptosis (Fig. 5F-G). Consistent with *in-vitro* experiments, immunoblotting reviewed that anti-apoptosis BCL-2 was up-regulated by C12orf48 over-expression and partly reversed by concurrent C12orf48-PCBP1 over-expression. While the pro-apoptosis BAX and cleaved caspase 3 were down-regulated by C12orf48 over-expression and rescued by PCBP1. Besides, the expression of EMT-related proteins was also altered downstream C12orf48. Compared with the control group, the expression of Vimentin and N-cadherin was up-regulated in C12orf48 over-expression group, while the expression of E-cadherin was down-regulated. And the altered expression of EMT-related proteins partially reversed by PCBP1 over-expression (Fig. 5H-I).

## Discussion

In this study, we sort to clinical, cellular, and animal experiments, to explore the critical role and molecular basis of altered C12orf48 expression in gastric cancer. The results provided evidence that C12orf48 was more frequently over-expressed in gastric cancer tissues and associated with poorer prognosis. And cancer growth and metastasis were inhibited when C12orf48 down-regulated both *in-vitro* and *in-vivo*. More importantly, we found that PCBP1, a novel tumor suppressor gene, is inhibited by C12orf48 in gastric cancer. Intended over-expression of PCBP1 abolishes the effects of C12orf48 both in vitro and in vivo. Together, these results unveiled that C12orf48-PCBP1 signaling contributes to gastric carcinogenesis.

PARP1, thought to be strongly related to a series of cellular processes involving DNA repair, transcriptional, and posttranscriptional modulation of gene expression, is an essential nuclear protein that plays a pivotal role in various DNA repair pathways and in the maintenance of genomic stability [11]. PARP1 directly or indirectly interacts with diverse oncogenic proteins or transcription factors, thus salvaging cancer cells from apoptosis and further modulating carcinogenesis. Recent years have witnessed various studies concerning PARP inhibitors that boasted a promising anticancer therapeutic effect, despite PARP inhibitor resistance has gradually been in the spotlight. Simultaneously, a heated direction regarding alterations in the levels of PARP1 activity and expression has been emerging, which sheds light on the potential tumorigenic capacity of PARP1 regulatory proteins [12]. Besides, studies have discovered some delicate relationship between PARP1 and EMT. It is well-known that the loss of E-cadherin is one of the two crucial steps in EMT, which can be affected via E-cadherin regulatory axis where PARP1 plays a role. The disruption of tight junctions (TJ) is the other significant process that contributes to EMT, highlighting the loss of cohesive property that prompts invasion and metastasis of cancer cells. Thereinto, transforming growth factor β (TGF-β), capable of downregulating certain TJ proteins as well as E-cadherin, induces EMT from another prospective. It has been reported that PARP1 functional inhibition affects EMT via the TGF-β signaling in a manner [13], which plausibly gives a hint that any measures leading to the hindrance of PARP1 overexpression could possibly be linked with TGF-β signaling pathway.

PARP1-binding protein, encoded by gene C12orf48, is one of the PARP1 regulatory proteins. C12orf48 is capable of interacting with PARP1 directly, which can strengthen the ability of PARP1 to repair DNA injuries [3]. In other words, C12orf48 might have an important bearing on the protection of cancer cells from apoptosis under the circumstances of DNA damage or cellular stresses on the basis of its positive regulation towards PARP1 activity. Moreover, C12orf48 was found co-expressed with some fundamental mitotic genes that were highly associated with carcinogenesis in cell cycle [14]. Intriguingly, a previous study showed that in certain malignancies such as myeloid leukemia cells [5], hepatocellular cancer [6] and pancreatic cancer [7], the upregulation of C12orf48 expression has been detected, suggesting that C12orf48 could be a potential inhibiting target for novel therapeutic anticancer treatment. Very recently, a study showed that overexpression of C12orf48 correlated with chemotherapy resistance in breast cancer [15]. Hence, taken together, we believe that high expression of C12orf48 could be a prognostic marker in cancer cells, and knockdown of C12orf48 might be a solution to attenuate the ability of PARP1 and preserve genomic stability.

PCBP1, belonging to the heterogeneous nuclear ribonucleoprotein (hnRNP) family and sharing 82% amino acid similarity with PCBP2, is an RNA-binding protein thought to have been derived from a fully processed PCBP2 mRNA by retrotransposition, which is ubiquitously expressed in mRNA regulation. It is a novel tumor-suppressive splicing factor that regulates the process of pre-mRNA, mRNA stabilization and translation [9]. By binding specifically to elements of target mRNAs with AU-rich elements or U-rich elements located in 3’-untranslated regions, PCBP1 regulates diverse gene expression [16]. Studies showed that PCBP1 protein was significantly downregulated in various primary and metastatic cancers, including gastric cancers [17], while its mRNA level remained unchanged in most cancer tissues [9]. And cell spreading, more specifically, the migration and metastasis of cancer cells was found stimulated when PCBP1 was inhibited. Moreover, the tumorigenicity of cancer cells, including gastric cancers, was attenuated once PCBP1 was overexpressed [16]. In the present study, we demonstrated that intended over-expression of PCBP1 attenuated the tumor growth and metastasis mediated by C12orf48, suggesting that altered PCBP1 expression is responsible for regulating cellular processes downstream C12orf48. Simultaneously, better outcome of patients with certain cancers was reported in the presence of PCBP1 overexpression. Besides, PCBP1 was considered as a mediator that played a role in TGF-β-induced EMT in gall bladder carcinomas [18]. Recent studies indicated that the process of EMT existing in a variety of cancer cells could be triggered by the phosphorylation of PCBP1 at Ser43 by Akt2 upon TGF-β stimulation [19]. Furthermore, we found that PCBP1 overexpression contributes to up-regulation of mesenchymal marker vimentin while down-regulation of epithelial marker E-cadherin both *in-vitro* and *in-vivo*, which reversed C12orf48-mediating EMT in gastric cancer. From above, these studies strongly suggested that PCBP1 was a tumor suppressor and had the capacity of attenuating tumorigenicity of cancer cells.

## Conclusion

In conclusion, the present study elucidated that depletion of C12orf48 attenuates growth and metastasis processes of gastric cancer via up-regulation of PCBP1. The result highlights that C12orf48-PCBP1 axis contributes to gastric carcinogenesis and provide new insights into gastric cancer treatment by targeting C12orf48-PCBP1 axis.

## Supplementary Information

**Additional file 1.**

## Data Availability

The datasets used and/or analysed during the current study are available in the website: http://gepia.cancer-pku.cn/detail.php?gene=PARPBP, STAD datasets.

## Abbreviations

ATCC: American Type Culture Collection
EMT: Epithelial mesenchymal transition
FBS: Fetal bovine serum
GEPIA: Gene Expression Profiling Interactive Analysis
GTEx: Genotype-Tissue Expression
IHC: Immunohistochemistry
PARP1: Poly(ADP-Ribose) Polymerase 1
PARPBP: PARP1 binding protein
PCBP: Poly r(C)-Binding Protein
mOS: Median overall survival
O.D.: Optical density
OS: Overall survival
qRT-PCR: Quantitative Real Time PCR
SD: Standard deviation
shRNA: Short hairpin RNA
TCGA: The Cancer Genome Atlas
TGF-β: Transforming growth factor β
TMA: Tissue microarray
Tunel: TdT-mediated dUTP Nick-End Labeling
UICC: Union for International Cancer Control
