# Supplementary figures and images for: Depletion of C12orf48 inhibits gastric cancer growth and metastasis via up-regulating Poly r(C)-Binding Protein (PCBP) 1
Source: BMC Cancer. 2022 Jan 31;22:123. doi: 10.1186/s12885-022-09220-0 (PMC8802463; doi:10.1186/s12885-022-09220-0)

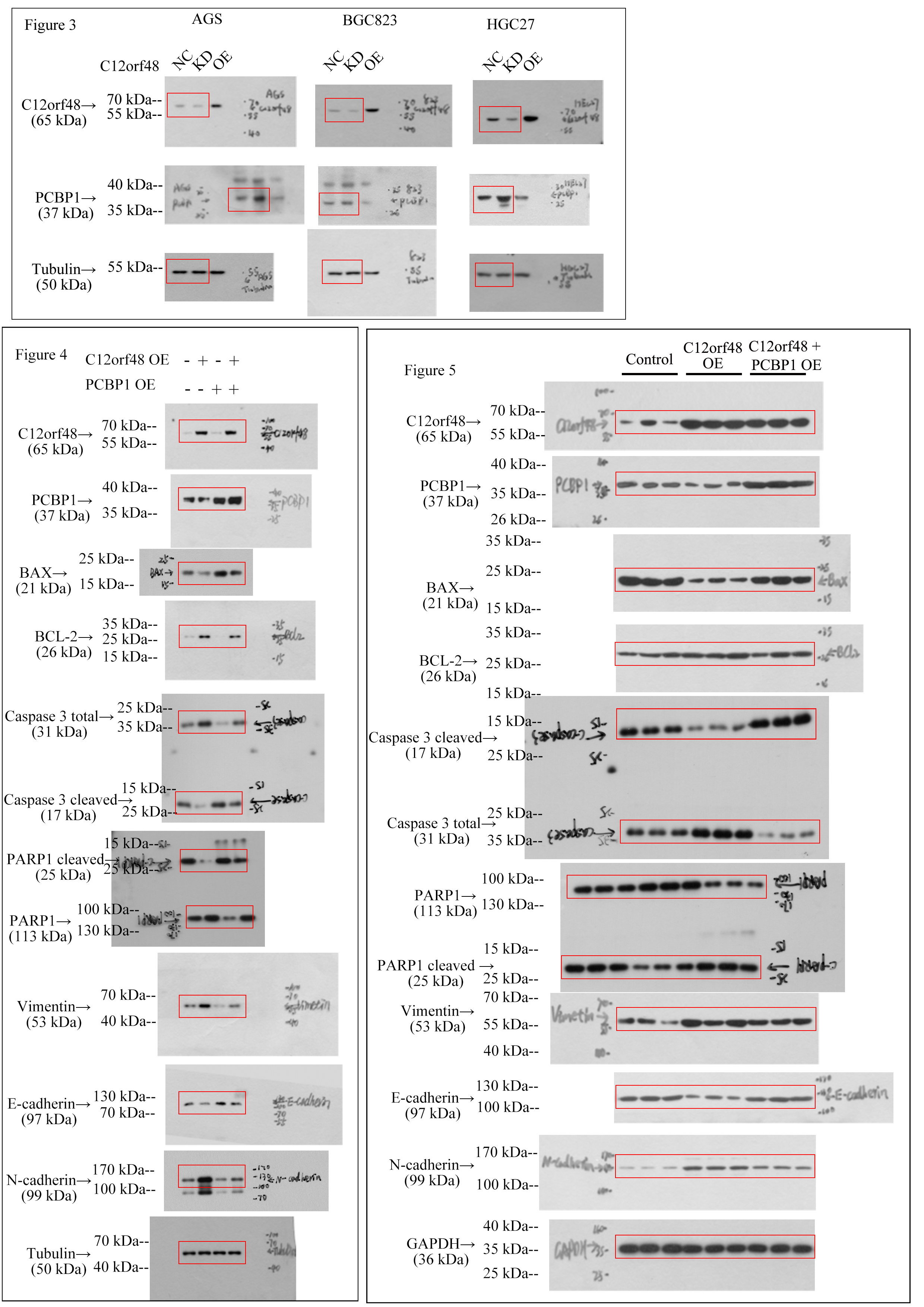

Supplement: Supplementary file 1 — Additional file 1. [file 12885_2022_9220_MOESM1_ESM.tif]
